# Meta-Analysis of Asymmetric Dimethylarginine Concentrations in Rheumatic Diseases

**DOI:** 10.1038/s41598-019-41994-5

**Published:** 2019-04-01

**Authors:** Gian Luca Erre, Arduino Aleksander Mangoni, Floriana Castagna, Panagiotis Paliogiannis, Ciriaco Carru, Giuseppe Passiu, Angelo Zinellu

**Affiliations:** 1 UOC Reumatologia, Dipartimento di Medicina Clinica e Sperimentale, Azienda Ospedaliero-Universitaria di Sassari and Università di Sassari, Sassari, Italy; 2 Department of Clinical Pharmacology, College of Medicine and Public Health, Flinders University and Flinders Medical Centre, Adelaide, Australia; 3 Dipartimento di Scienze Biomediche, Università degli Studi di Sassari, Sassari, Italy

## Abstract

Raised circulating concentrations of asymmetric dimethylarginine (ADMA), an endogenous inhibitor of nitric oxide synthase (NOS), have been reported in several rheumatic diseases (RDs). However, the strength of this relationship is unclear. Therefore, the aim of this systematic review and meta-analysis was to evaluate the magnitude and the robustness of the association between ADMA concentrations and RDs. We calculated standardized mean differences (SMD, with 95% confidence intervals, CI). Study heterogeneity was evaluated by meta-regressions and sensitivity analyses according to type of RDs, conventional cardiovascular risk factors, inflammatory markers, and type of ADMA assessment methodology. Thirty-seven studies with a total of 2,982 subjects (1,860 RDs patients and 1,122 healthy controls) were included in our meta-analysis. Pooled results showed that ADMA concentrations were significantly higher in patients with RDs than in healthy controls (SMD = 1.27 µmol/L, 95% CI 0.94–1.60 µmol/L; p < 0.001). However, the between-studies heterogeneity was high. Differences in ADMA concentrations between controls and RDs patients were not significantly associated with inflammatory markers, increasing age, lipid concentrations, body mass index, blood pressure, or methodology used to assess ADMA. Furthermore, subgroup analysis showed no difference across RDs. This meta-analysis showed that, in the context of significant between-study heterogeneity, circulating concentrations of ADMA are positively related to RDs.

## Introduction

The term “systemic rheumatic diseases” (RDs) encompasses a broad spectrum of chronic inflammatory disorders of joints and internal organs that are characterized by tissue destruction, disability and increased cardiovascular and all-cause mortality. The dysregulation of innate and adaptive immunologic response, with overproduction of pro-inflammatory cytokines, represents the pathogenic hallmark of RDs^1^.

Compared with the general population, patients with RDs suffer from a significantly reduced life expectancy that is mainly due to atherosclerotic cardiovascular disease^2,3^. This excess of cardiovascular mortality, only partially predicted by conventional risk factors, has been linked to early endothelial dysfunction and accelerated arterial stiffening^2–4^. In fact, peripheral and central endothelial dysfunction, a measure of nitric-oxide (NO) availability and vasodilatory function, has been shown to be more prevalent in RDs than in the general population^5–7^.

A strong body of clinical and experimental evidence collected in the last decade has convincingly supported a potential pathogenetic role for the accumulation of asymmetric dimethylarginine (ADMA), an endogenous inhibitor of NO-synthase^8^, in the occurrence and progression of endothelial dysfunction, arterial stiffening, and atherosclerotic cardiovascular disease^9^. Moreover, studies in healthy subjects and different patient groups have confirmed the independent prognostic role of basal plasma ADMA concentrations on cardiovascular and all-cause mortality^10–12^. Therefore, raised circulating ADMA concentrations might be a surrogate measure of endothelial dysfunction, early atherosclerosis and increased risk of future cardiovascular events.

Increased circulating ADMA concentrations, initially reported in rheumatoid arthritis (RA) patients free of cardiovascular risk^13^, have also been observed in psoriatic arthritis (PsA)^14,15^, ankylosing spondylitis (AS)^16–20^, systemic lupus erythematosus (SLE)^21^, Sjogren’s syndrome (SSj)^22^, systemic sclerosis (SSc)^23–29^ and Behçet’s disease (BD)^30–34^.

However, despite being largely investigated, the association between ADMA concentrations and RDs has generally been studied in populations with relatively small sample size. Therefore, we performed a meta-analysis of all relevant studies to define the magnitude and the robustness of the relationship between ADMA concentrations and RDs.

## Materials and Methods

### Search strategy, eligibility criteria, and study selection

A systematic search of publications in the PubMed, Web of Science and Scopus databases, from inception to March 2018, was performed using the following keywords and terms, and their combination: ‘ADMA AND (rheumatic diseases OR rheumatoid arthritis OR psoriatic arthritis OR ankylosing spondylitis OR systemic lupus erythematosus OR systemic sclerosis OR Sjogren’s syndrome OR connective tissue diseases OR vasculitis OR Behçet’s disease)’. In addition, references of the filtered studies were manually checked for potentially missing eligible studies. Two researchers (GLE and AZ) independently screened the abstracts and reviewed the full articles; disagreement between the investigators, when present, was resolved by a third researcher (PP). Inclusion criteria were: (i) assessment of ADMA, (ii) only adult participants, (iii) comparison of healthy subjects with patients with RDs (case-control design), (iv) English language and (vi) full-text publications.

A pre-established protocol, including methods for the analysis, was followed: in particular, we investigated the difference in ADMA concentrations stratifying for the following subgroups (i) RD subtype (e.g. RA vs SSc vs PsA, etc.) (ii) connective tissue diseases (CTD) vs non-CTD, (iii) autoimmune conditions (SLE, RA, SSc and SSj) vs conditions with mixed (autoimmune and inflammatory) features (PsA, AS) and autoinflammatory conditions (BD and Familial Mediterranean Fever, FMF), (iv) biological sample (serum vs plasma) and (v) type of laboratory test (e.g. liquid chromatography-LC vs ELISA).

Where available, we extracted demographic, anthropometric, clinical and laboratory data from the selected articles and presented them in table format.

Quality of studies was assessed with the Newcastle–Ottawa Scale (NOS)^35^. Meta-regression analysis was carried out to investigate potentially influencing factors on ADMA% difference.

### Statistical analysis

Standardized mean differences (SMD) were used to create forest plots of continuous data and to assess differences in ADMA concentrations between healthy controls and patients with RDs. A p < 0.05 was considered statistically significant, and 95% confidence intervals (CIs) were reported. In two studies^22,23^ the mean and standard deviation were inferred from median and IQR as previously reported^36^, while in two further studies^30,37^ the mean and standard deviation were inferred from median and range as previously reported by Hozo *et al*.^38^.

Heterogeneity of SMD across studies was tested with the Q statistic (significance level at p < 0.10). The I^2^ statistic was also calculated (I^2^ < 25%, no heterogeneity; I^2^ between 25% and 50%, moderate heterogeneity; I^2^ between 50% and 75%, large heterogeneity; and I^2^ > 75%, extreme heterogeneity)^39,40^. Statistical heterogeneity was defined as an I^2^ statistic value ≥ 50% (38). In analyses in which heterogeneity was high, a random-effects model was used.

The influence of each single study on effect size was evaluated by sequentially excluding one study at the time, through sensitivity analysis. Publication bias was assessed by means of Begg’s adjusted rank correlation test and Egger’s regression asymmetry test at the p < 0.05 level of significance^41,42^. Duval and Tweedie “trim and fill” procedure was performed to identify and correct for funnel plot asymmetry resulting from publication bias^43^. The aim of this method is to remove (trim) smaller studies responsible for funnel plot asymmetry, use the trimmed funnel plot to evaluate the true centre of the funnel, then replacing the omitted studies with the new calculated “missing” studies around the centre (filling). This allows to estimate the number of missing studies and recalculates the effect size including the filled studies. Statistical analysis was carried out using MedCalc for Windows, version 15.4 64 bit (MedCalc Software, Ostend, Belgium) and Stata 14 (STATA Corp., College Station, TX, USA)

This meta-analysis was performed according to PRISMA statements.

## Results

### Literature Search and Study Selection

**Figure 1:**
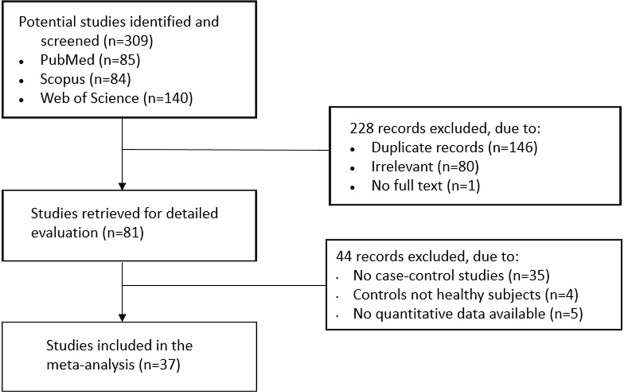
Flowchart of study selection.

A flowchart summarizing the screening process is described in Fig. 1. We initially detected 309 potentially relevant studies (PubMed n = 85, Scopus n = 84 and WOS n = 140). Two hundred and twenty eight studies were excluded after the initial screening because they were either duplicates (n = 146), irrelevant (n = 81) or not available in full text (n = 1).

After a full-text review of 81 articles, 44 were rejected because they did not meet the inclusion criteria. Thus, 37 studies were included in the meta-analysis^13–17,19–34,37,44–57^. The characteristics of these studies, published between 2006 and 2018, are summarized in Table 1.

**Table 1:** Summary of the studies on controls vs subjects with rheumatic diseases included in the meta-analysis.

First Author, cit Year, Country					CTRLs				RDs			
	Disease	Sample	ADMA Assay	NOS	n	Age Mean or Median	Gender (M/F)	ADMA Mean ± SD	n	Age Mean or Median	Gender (M/F)	ADMA Mean ± SD
Dooley *et al*.^24^, UK	SSc	s	ELISA	5	25	48.0	6/19	0.67 ± 0.04	45	51.5	12/33	0.88 ± 0.09
Sahin *et al*.^31^, Turkey	BD	p	ELISA	7	30	—	—	0.86 ± 0.56	60	31.0	39/21	3.18 ± 1.21
Surdacki *et al*.^13^, Poland	RA	p	LC-MS	7	20	45.0	3/17	0.40 ± 0.07	30	47.5	5/25	0.49 ± 0.07
Terekeci *et al*.^45^, Turkey	FMF	s	ELISA	5	23	20.5	23/0	0.31 ± 0.07	38	20.5	38/0	0.54 ± 0.10
Wipff *et al*.^25^, France	SSc	s	ELISA	8	48	59.4	8/40	0.89 ± 0.30	187	55.9	30/157	0.86 ± 0.35
Blaise *et al*.^26^, France	SSc	p	LC-MS	6	24	52.0	3/21	0.62 ± 0.12	39	52.0	4/35	0.68 ± 0.12
Sari *et al*.^16^, Turkey	AS	s	ELISA	5	38	36.4	27/11	0.9 ± 0.9	48	38.6	36/12	1.6 ± 1.0
Turiel *et al*.^46^, Italy	RA	p	LC-FLR	8	25	50.5	4/21	0.58 ± 0.07	25	52.0	4/21	0.66 ± 0.07
Dimitroulas *et al*.^29^, Greece	SSc	s	ELISA	8	25	—	—	0.25 ± 0.13	52	55.7	1/51	0.34 ± 0.18
Atzeni *et al*.^14^, Italy	PsA	p	LC-FLR	6	35	55.4	19/16	0.48 ± 0.07	22	54.9	12/10	0.71 ± 0.07
Erre *et al*.^17^, Italy	AS	p	CE-UV	8	17	38.0	10/7	0.54 ± 0.07	17	39.0	10/7	0.65 ± 0.10
Karaoglan *et al*.^48^, Turkey	RA	s	LC-FLR	7	18	46.3	2/16	0.4 ± 0.16	18	49.4	2/16	0.52 ± 0.19
Saadany *et al*.^21^, Egypt	SLE	s	ELISA	6	20	31.4	0/20	0.58 ± 0.05	30	30.1	0/30	0.68 ± 0.02
Topal *et al*.^47^, Turkey	FM	s	ELISA	6	23	40.0	4/19	0.58 ± 0.30	25	40.7	2/23	0.78 ± 0.31
Aydin *et al*.^32^, Turkey	BD	s	ELISA	7	24	34.5	7/17	0.55 ± 0.09	49	34.1	13/36	1.01 ± 0.32
Di Franco *et al*.^49^, Italy	RA	s	ELISA	8	20	—	—	0.41 ± 0.02	20	51.0	7/13	0.55 ± 0.03
Kwaśny-Krochin *et al*.^50^, Poland	RA	p	LC-FLR	8	50	56.0	7/43	0.46 ± 0.05	46	57.0	7/39	0.58 ± 0.08
Sandoo *et al*.^51^, UK	RA	s	ELISA	6	29	42.0	8/21	0.37 ± 0.07	67	56.0	19/48	0.47 ± 0.13
Korkosz *et al*. (a).^18^, Poland	AS	s	ELISA	6	23	32.3	11/12	0.65 ± 0.19	78	35.7	—	0.64 ± 0.19
Korkosz *et al*. (b).^18^, Poland	RA	s	ELISA	6	23	32.3	11/12	0.65 ± 0.19	29	41.0	—	0.77 ± 0.20
Ozuguz *et al*.^33^, Turkey	BD	s	ELISA	7	20	37.0	8/12	0.10 ± 0.04	40	39.6	15/25	0.9 ± 0.7
Pamuk *et al*.^37^, Turkey	FMF	p	ELISA	6	18	35.5	11/7	2.76 ± 0.69°	40	31.0	21/19	2.56 ± 0.81°
Turiel *et al*.^23^, Italy	SSc	p	LC-FLR	8	20	55.4	6/14	0.56 ± 0.05*	20	53.0	2/18	0.86 ± 0.07*
Vatansev *et al*.^53^, Turkey	RA	s	LC-FLR	5	34	46.8	19/15	3.24 ± 1.44	92	43.8	13/79	4.6 ± 2.64
Atzeni *et al*.^22^, Italy	SSj	p	LC-FLR	7	22	59.3	6/16	0.55 ± 0.04*	22	60.1	6/16	0.82 ± 0.04*
Ciurzyński *et al*.^27^, Poland	SSc	s	ELISA	7	21	49.3	3/18	0.46 ± 0.09	111	54.2	10/101	0.45 ± 0.14
Klimek *et al*.^53^, Poland	RA	p	ELISA	6	29	31.7	16/13	0.67 ± 0.18	29	41.0	7/22	0.77 ± 0.20
Öztürk *et al*.^30^, Turkey	BD	s	LC-FLR	6	34	33.3	19/15	3.22 ± 0.72°	34	37.5	19/15	2.25 ± 0.97°
Berg *et al*.^19^, Norway	AS	p	LC-FLR	6	134	52.7	78/56	0.49 ± 0.08	151	49.2	92/59	0.54 ± 0.12
Yilmazer *et al*.^15^, Turkey	PsA	s	ELISA	7	20	40.3	9/11	0.63 ± 0.19	20	41.4	5/15	0.54 ± 0.15
Yuksel *et al*.^34^, Turkey	BD	s	ELISA	7	35	30.1	21/14	1.96 ± 0.79	36	32.1	19/17	3.2 ± 0.54
Akgol *et al*.^54^, Turkey	RA	s	LC-FLR	7	30	53.0	8/22	0.35 ± 0.11	40	51.4	8/32	0.44 ± 0.15
Erre *et al*.^55^, Italy	RA	p	ELISA	8	30	54.1	2/28	0.9 ± 0.30	30	55.0	2/28	1.0 ± 0.3
Şentürk *et al*.^56^, Turkey	RA	—	ELISA	6	29	46.1	2/27	0.29 ± 0.08	40	43.9	1/39	0.55 ± 0.20
Silva *et al*.^28^, Portugal	SSC	s	ELISA	7	34	47.1	5/29	0.38 ± 0.08	77	52.0	5/72	0.48 ± 0.10
Inci *et al*.^20^, Turkey	AS	s	ELISA	6	40	30.0	35/5	0.53 ± 0.16	60	32.2	53/7	0.75 ± 0.19
Ozalper *et al*.^57^, Turkey	FMF	s	ELISA	6	35	24.7	35/0	1.43 ± 0.49^§^	57	24.3	57/0	1.48 ± 1.06^§^
Radhakutty *et al*.^44^, Australia	RA	p	LC-MS	8	20	63.0	4/16	0.48 ± 0.01	36	65.0	12/24	0.55 ± 0.03

NOS: Newcastle–Ottawa quality assessment scale for case-control studies (number represent stars). S, serum; p, plasma; LC, liquid chromatography;

MS, mass spectrometry; FLR, fluorimetric; CE-UV, capillary electrophoresis UV detection. *Mean and standard deviation were estimated from the median and IQR. °Mean and standard deviation were estimated from the median and range. ^§^Calculated from ng/dL. CTRLs, controls. RDs, rheumatic diseases. SSc, systemic sclerosis; BD, Behcet’s disease. RA, rheumatoid arthritis; FMF, familial Mediterranean fever; SSc, systemic sclerosis; AS, ankylosing spondylitis; PsA, psoriatic arthritis; SLE, systemic lupus erythematosus; FM, fibromyalgia.

### ADMA and RDs

A total of 1,860 RDs patients (41% males) and 1,122 healthy controls (34% males) were evaluated. Overall, the mean age of participants across all studies was 44.6 years both in RDs patients and in controls (Tables 1 and 2).

**Table 2:** Pooled standard mean difference according to rheumatic disease type.

Disease type	Overall Effect		Heterogeneity		N° of studies
	*SMD (95% CI)*	*p-value*	*I*^2^, %	*p-value*	
RA	1.28 (0.83–1.72)	<0.001	87.9	<0.001	13
SSc	1.24 (0.41–2.07)	0.003	94.8	<0.001	7
AS	0.69 (0.27–1.12)	0.001	80.3	<0.001	5
BD	1.20 (−0.04–2.45)	0.058	96.1	<0.001	5
FMF	0.77 (−0.73–2.27)	0.317	95.6	<0.001	3
PsA	1.37 (−2.38–5.11)	0.474	98.1	<0.001	2

SMD, standard mean difference. RA, rheumatoid arthritis; SSc, systemic sclerosis; AS, ankylosing spondylitis; BD, Behcet’s disease; FMF; familial Mediterranean fever; PsA, psoriatic arthritis.

**Figure 2:**
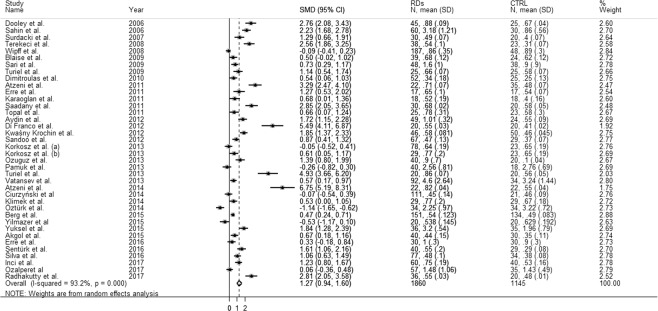
The pooled standard mean difference (SMD) for asymmetric dimethylarginine (ADMA) concentrations with 95% confidence intervals for eligible studies.

The forest plot for ADMA concentrations in RDs patients and controls is shown in Fig. 2. Due to the extreme heterogeneity between studies (I^2^ = 93.2%, p < 0.001), random-effects models were used to perform the analysis. Pooled results showed that ADMA concentrations were significantly higher in patients with RDs (SMD = 1.27 µmol/L, 95% CI 0.94–1.60 µmol/L; p < 0.001).

**Figure 3:**
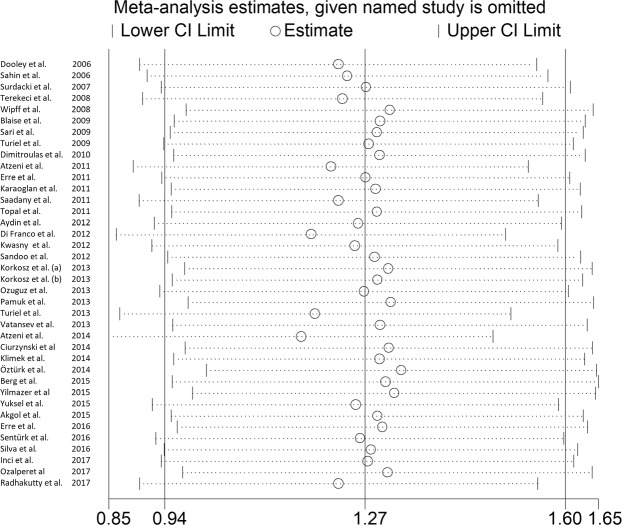
Sensitivity analysis of studies examining asymmetric dimethylarginine (ADMA) concentrations in rheumatic diseases (RDs).

Sensitivity analysis showed that the effect size was not modified when any single study was in turn removed (effect size ranged between 1.17 µmol/L and 1.33 µmol/L, Fig. 3).

**Figure 4:**
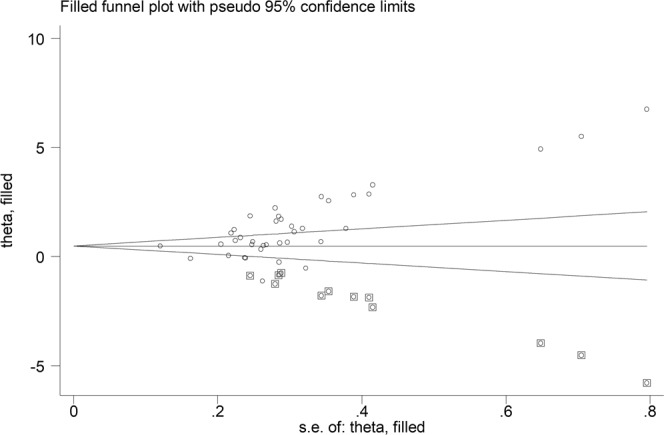
Funnel plot of eligible studies on dimethylarginine (ADMA) concentrations in rheumatic diseases (RDs).

Since the Begg’s (p < 0.001) and Egger’s tests (p < 0.001) evidenced a significant publication bias, we applied the trim-and-fill method to correct the results. Twelve potential missing studies were added on the left side of the funnel plot to ensure symmetry (Fig. 4). The adjusted SMD was decreased, but remained significant (0.51 µmol/L, 95% CI 0.15–0.88 µmol/L, p = 0.006).

To explore possible sources of heterogeneity, we investigated differences in SMD across RDs (Table 2). Extreme heterogeneity was observed in all disease types (Table 2). RA, SSc and AS but not BD, FMF, and PsA patients showed significantly higher SMD when compared to healthy controls. However, no significant differences in SMD values were observed between different disease types by meta-regression analysis (p > 0.05).

**Figure 5:**
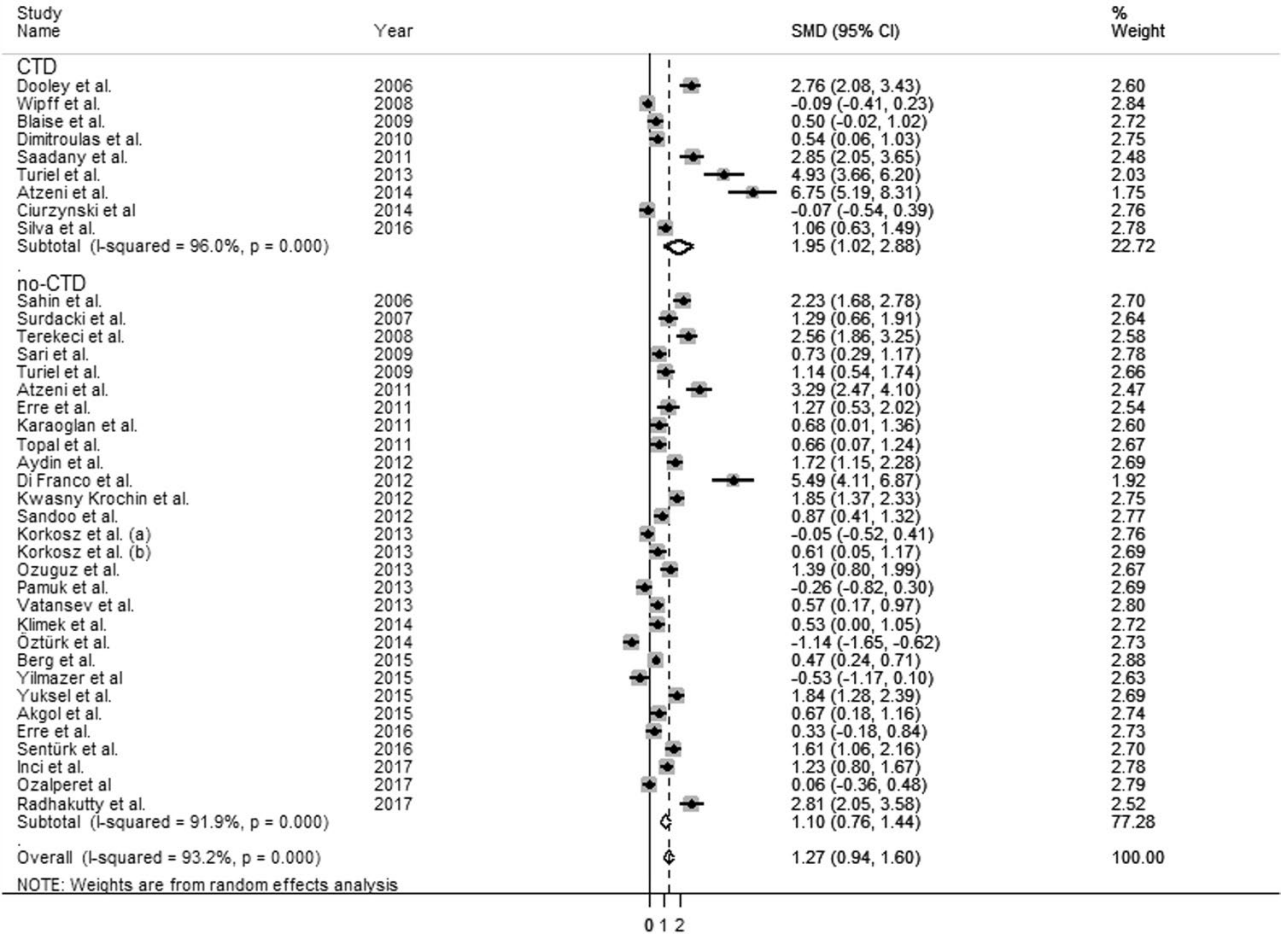
Forest plot depicting the standard mean (SMD) of dimethylarginine (ADMA) concentrations in connective tissue (CTD) *vs* no-CTD rheumatic diseases. CTD: Systemic lupus erythematosus (SLE), Systemic sclerosis (SS), Rheumatoid arthritis (RA), Sjogren’s syndrome (SSj).

Moreover, the SMD values in studies of participants with connective tissue diseases (CTD) (1.95 µmol/L, 95% CI 1.02–2.88 µmol/L, p < 0.0001; I^2^ = 96.0%, p < 0.0001) were higher than that in participants with no-CTD disease (1.10 µmol/L, 95% CI 0.76–1.44 µmol/L, p < 0.0001; I^2^ = 91.9%, p < 0.0001) although the difference was not significant by meta-regression analysis (t = −1.40, p = 0.17, Fig. 5). Classification of RDs according to CTD phenotype did not influence heterogeneity across studies.

**Figure 6:**
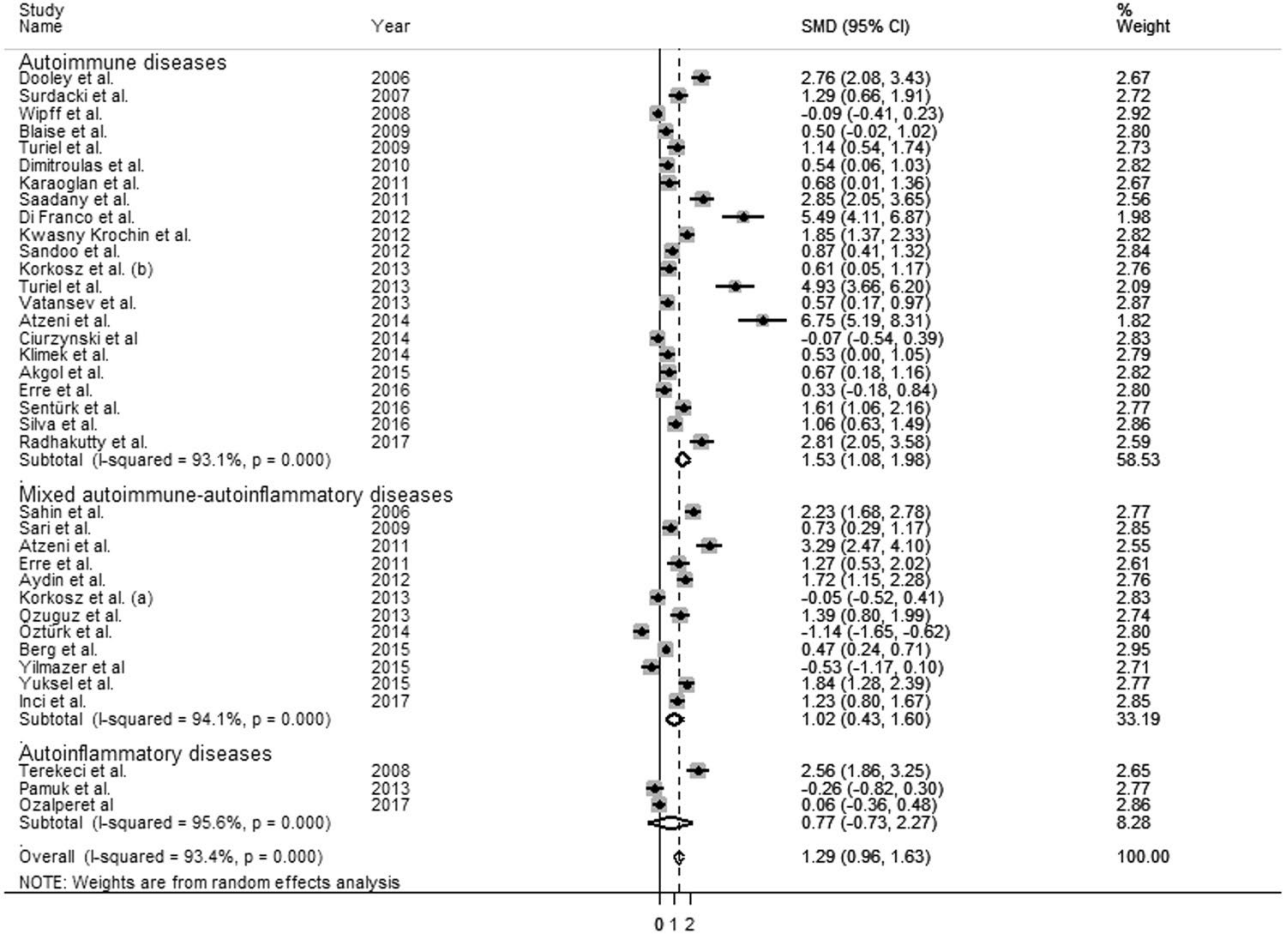
Forest plot depicting the standard mean (SMD) of dimethylarginine (ADMA) concentrations in autoimmune (Systemic lupus erythematosus-SLE, Systemic sclerosis-SS, Rheumatoid arthritis-RA, and Sjogren’s syndrome-SSj) *vs* mixed autoimmune-autoinflammatory (Behcet’s disease-BD, psoriatic arthritis-PsA, and ankylosing spondylitis-AS) *vs* autoinflammatory (Familial Mediterranean fever (FMF).

As reported in in Fig. 6, there was no significant difference in SMD values in RDs patients affected by pure autoimmune diseases (1.53 µmol/L, CI 1.08, 1.98 µmol/L, p < 0.0001; I^2^ = 93.1%, p < 0.0001) when compared with RDs patients with mixed (autoimmune and autoinflammatory) diseases (1.02 µmol/L, CI 0.43, 1.60 µmol/L, p = 0.006; I2 = 94.1%, p = 0.0001) or with autoinflammatory diseases (0.77 µmol/L, CI −0.73, 2.27 µmol/L, p = 0.317; I2 = 95.6%, p < 0.0001). Similarly, heterogeneity was not influenced by disease sub-groups.

We then investigated C-reactive protein (CRP), erythrocyte sedimentation rate (ESR), systolic blood pressure (SBP), diastolic blood pressure (DBP), lipid concentrations, body mass index (BMI), age, sex, and disease duration (DD) as possible contributors to between-study variance. However, none of these variables was found significantly related to pooled SMD by meta-regression analysis [CRP (t = 0.72, p = 0.477), ESR (t = 0.73, p = 0.475), SBP (t = 0.63, p = 0.537), DBP (t = 1.11, p = 0.29), DD (t = 0.93, p = 0.362), total cholesterol (t = −1.71, p = 0.105), LDL (t = −1.05, p = 0.305), HDL (t = −1.56, p = 0.138), TG (t = 0.86, p = 0.399), BMI (t = −0.02, p = 0.981), age (t = 1.47, p = 0.152) and sex (t = −0.19, p = 0.850)].

**Figure 7:**
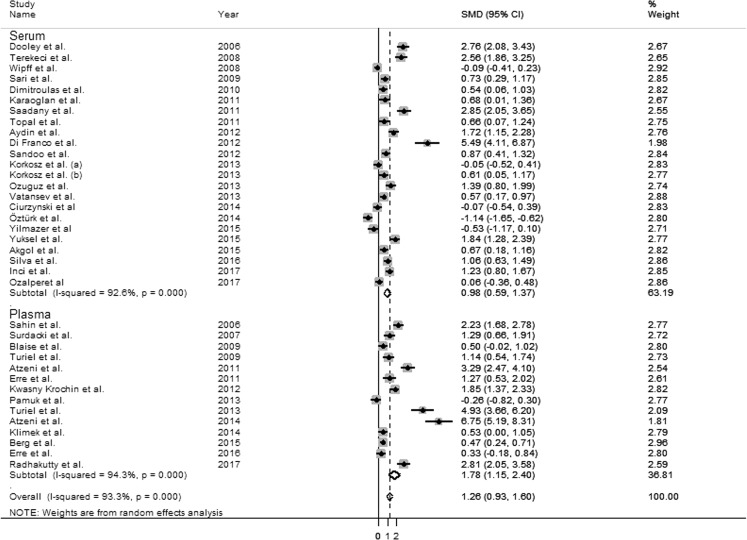
Forest plots depicting the standard mean (SMD) of dimethylarginine (ADMA) concentrations in rheumatic diseases (RDs) according to the biological matrix tested (serum *vs* plasma).

Finally, we considered whether methodological factors, as biological sample type (serum vs plasma) or detection method (LC vs ELISA) may contribute to heterogeneity. The SMD from studies measuring ADMA in plasma (1.78 µmol/L, 95% CI 0.93–1.60 µmol/L, p < 0.0001; I^2^ = 94.3%, p < 0.0001) was higher than that in studies measuring ADMA in serum (0.98 µmol/L, 95% CI 0.59–1.37 µmol/L, p < 0.0001; I^2^ = 92.6%, p < 0.0001, Fig. 7), but the difference was not statistically significant (t = −1.53, p = 0.135).

**Figure 8:**
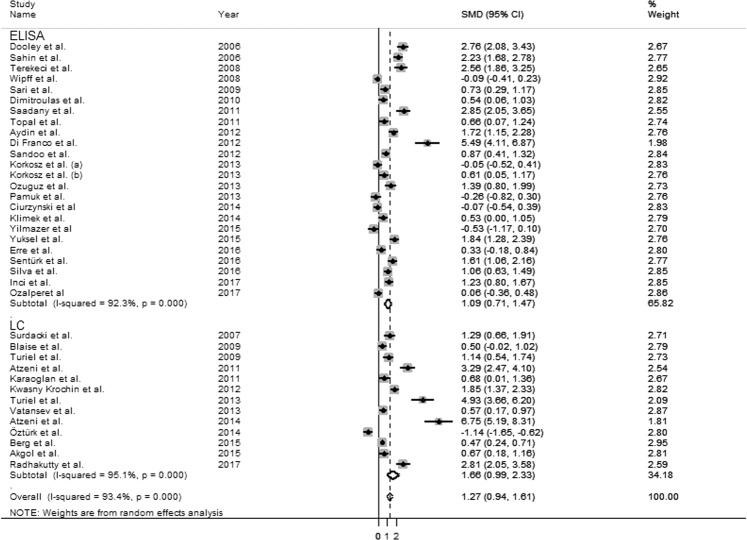
Forest plots depicting the standard mean (SMD) of dimethylarginine (ADMA) concentrations in rheumatic diseases (RDs) according to analytical techniques (liquid chromatography-LC *vs* ELISA).

Furthermore, the SMD from studies measuring ADMA by LC was higher (1.66 µmol/L, 95% CI 0.99–2.33 µmol/L, p < 0.0001; I^2^ = 95.1%, p < 0.0001) than that of studies measuring ADMA by ELISA (1.09 µmol/L, 95% CI 0.71–1.47 µmol/L, p < 0.0001; I^2^ = 92.3%, p < 0.0001, Fig. 8) without a statistically significant difference (t = 1.06, p = 0.296). Heterogeneity in sub-groups was extremely high.

## Discussion

Besides their effect on the vascular tone, high circulating concentrations of ADMA have been reported to promote oxidative stress^58^ vascular inflammation^59^ and smooth muscle cell proliferation^60,61^. Therefore, ADMA is a candidate pathogenetic factor for oxidative stress-related endothelial dysfunction, accelerated atherosclerosis and vascular remodelling process^62^ occurring in RDs.

However, the results of studies reporting the association between ADMA and RDs have been contradictory: although most studies demonstrated a significant association between ADMA and RDs^13,14,16,17,19–24,26,28,29,31–34,44–50,52–56,63^, other studies failed to report a significant relationship^15,18,25,27,30,37,57^. The different results might be at least partially explained by the small sample size of each study.

Therefore, we performed a systematic review and meta-analysis to evaluate the strength of the association between circulating ADMA concentrations and RDs. Our analysis, including data from 37 studies with a total of 2,982 subjects, suggested that RDs are associated with significantly higher concentrations of circulating ADMA.

However, we found substantial heterogeneity in studies estimating ADMA in RDs; Therefore, to explore in detail any potential sources of heterogeneity we presented data on a wide range of subgroups based on rheumatic disease phenotype and methodological aspects (Figs 5–8).

First, according to a pre-defined protocol, a subgroup analysis according to type of RDs was performed to identify the source of heterogeneity: other than expected, inclusion in the meta-analysis of different RDs did not account for the large heterogeneity reported in the pooled analysis; Even if SMD was higher in studies including RA, SSc and AS than in studies including BD, FMF and PsA (Table 2) this difference was not statistically significant across studies by meta-regression analysis.

We were also interested to understand whether specific pathogenetic and clinical features of RDs were associated with significant between studies heterogeneity. Therefore, according to pre-specified sub-group analyses, we stratified RDs in CTD vs no-CTD. However, this classification of RDs was not able to explain the large heterogeneity observed in the pooled analysis.

Moreover, according to Mc Goonagle *et al*.^64^, we stratified RDs in autoimmune diseases (e.g RA, SLE, and SSc), diseases with mixed autoimmune and autoinflammatory features (e.g, diseases associated with HLA-B27 and other MHC class I epitopes, such as AS, PsA and BD^65,66^) and autoinflammatory diseases (e.g FMF). However, the difference in autoimmune vs autoinflammatory features did not account for the observed heterogeneity.

A significant association between raised circulating ADMA concentrations and traditional atherosclerotic risk factors (hypercholesterolemia, hypertriglyceridemia, and hypertension) have been reported^67–69^ suggesting that ADMA is causally involved in the pathophysiology of atherosclerosis.

Interestingly, in our study, cardiovascular risk factors did not modify the ADMA SMD. These results question the currently accepted hypothesis that raised circulating ADMA concentrations, a potential measure of the presence of co-existing cardiovascular risk factors, may be associated with increased morbidity and mortality for cardiovascular events in RDs. Therefore, prospective studies with larger sample sizes are warranted to further define the significance of the relationship between ADMA and conventional atherosclerotic risk factor and the independent role of ADMA in the development of atherosclerotic disease in RDs.

Biomarkers of systemic inflammatory burden (such as CRP and ESR) have been shown to be correlated to circulating ADMA concentrations in the general population and in RDs^17,59,70^. However, in our meta-analyses, we found no significant association between ESR, CRP, disease duration and ADMA concentrations: the cross-sectional design and the small sample size of studies included in this meta-analysis may partly explain this finding.

We also performed two additional pre-specified meta-analyses to evaluate between-group differences according to the analytical techniques used and the biological matrix tested as a potential source of heterogeneity.

To date, preferred methods for the determination of plasma and serum ADMA concentrations are LC and ELISA. However, it is still a matter of debate whether these two methods are comparable to each other^71–73^. A recent meta-analysis reported plasma ADMA concentrations in healthy subjects ranging from 0.34 to 1.10 μmol/l^73^. On the contrary, normal serum ADMA concentrations in healthy subjects are less well defined: a recent study reported a range of normality of serum ADMA concentrations of 0.43–0.96 μmol/^74^.

In our subgroup meta-analysis, we found no difference in ADMA SMD on the basis of the analytic method employed or biological matrix tested, suggesting that the large heterogeneity between studies is not explained by techniques variability. However, as reported in Table 1, the range of mean plasma and serum ADMA concentrations in CTRLs was extremely large (from 0.10 to 3.22 μmol/L) with reported mean values not falling within the expected range in a large number of studies^30,33,34,37,52,57,75^. We think that this finding might be related to variability in the selection of healthy controls, pre-analytical and analytical issues, potentially accounting for the large heterogeneity of pooled estimates in this meta-analysis.

Finally, we explored the substantial heterogeneity of pooled analysis by evaluating whether specific studies might influence the final results. After excluding each study in turn, the significance of SMD remained stable in every meta-analysis.

Moreover, according to Begg’s method and Egger’s test, our meta-analyses carry a significant publication bias. Thus, the trim and fill method was used to adjust for funnel plot asymmetry: in particular, 12 studies were added to balance the funnel plot. The adjusted SMD, even if decreased, remained significant. This indicated that publication bias did not affect the result of the meta-analysis.

This meta-analysis has some limitations; First, substantial heterogeneity of the included studies might decrease the significance of the results. Second, we only searched for studies written in English, an aspect that may have introduced publication bias. Third, most of the articles did not report the type of anti-inflammatory or anti-rheumatic drugs used and their effects on outcome. This last aspect is of both clinical and experimental relevance, considering that some prospective studies have reported a positive effect in reducing ADMA concentrations of immunosuppressive therapies^76,77^.

Strengths of this systematic review and meta-analysis on the topic of ADMA in RDs, performed on a relatively large and representative population, include the evaluation of ADMA in different disease phenotypes within the ‘rheumatic diseases’ umbrella and extensive analysis of the source of heterogeneity including pathogenetic and clinical features of RDs, analytical techniques and biological matrixes.

Moreover, this meta-analysis provided an estimated ‘effect size’ target of 1.27 umol/L for future interventional studies aiming at reducing ADMA in the population of patients with RDs.

## Conclusions

In summary, results from this meta-analysis show a significant positive association between elevated circulating ADMA concentrations and RDs.

The relatively high heterogeneity of the studies included in the meta-analysis, albeit extensively investigated with a number of additional analyses focused on clinical and demographic characteristics, analytical techniques, biological matrixes, and specific studies, requires further research to confirm, or refute, our findings.

## Data Availability

All data generated or analysed during this study are included in this published article.
